# Evaluation of an intelligent inpatient transportation system in a large tertiary hospital: a full implementation case review

**DOI:** 10.3389/fpubh.2026.1758228

**Published:** 2026-03-18

**Authors:** Chao Jiang, Huifen Zhu, Lin Deng, Yu Shu, Xixi Zhou, Lei Tong, Yaoying Zhou, Yanfei Ye, Min Cao

**Affiliations:** 1 Wound Repair Department, The Quzhou Affiliated Hospital of Wenzhou Medical University, Quzhou People's Hospital, Quzhou, Zhejiang, China; 2 Nursing Department, The Quzhou Affiliated Hospital of Wenzhou Medical University, Quzhou People's Hospital, Quzhou, Zhejiang, China; 3 Wenzhou Medical University, Wenzhou, China; 4 The Party and Government Comprehensive Office, The Quzhou Affiliated Hospital of Wenzhou Medical University, Quzhou People's Hospital, Quzhou, Zhejiang, China

**Keywords:** artificial intelligence, healthcare efficiency, hospital logistics, inpatient transportation, patient mobility, workflow optimization

## Abstract

**Introduction:**

Reliable inpatient transportation is a critical component of hospital operations and clinical workflow. As demand increases, traditional manual transport systems often struggle to meet service expectations. In response, our institution implemented an information technology–based inpatient transportation platform aimed at improving dispatch coordination and workflow integration.

**Methods:**

We conducted a retrospective, descriptive analysis of a hospital-wide implementation of an intelligent inpatient transportation platform integrated with the electronic medical record and mobile devices at Quzhou Hospital, China. The system was launched in June 2022. All completed inpatient transportation requests between August 31 and September 30, 2024 were included. Operational characteristics, transport time metrics, safety indicators, and user satisfaction were summarized without a comparator group.

**Results:**

A total of 15,543 completed transportation requests were analyzed. Mean transport times varied across departments, with the shortest average times in the ophthalmology and gynecology (34.78 and 34.94 min, respectively). The shortest intervals between scheduled and actual delivery times were observed in hand and foot surgery and in trauma orthopedics/emergency care (−44.86 and −34.91 min, respectively). No misidentification events were reported during the study period. Overall user satisfaction with the transportation service was 90%, with particularly high ratings for perceived service quality and timeliness.

**Conclusion:**

This retrospective implementation case study describes the operational performance, safety profile, and user-reported satisfaction associated with an intelligent inpatient transportation platform in a large tertiary hospital. While these descriptive findings suggest acceptable system functionality and feasibility, further prospective and comparative studies are needed to assess associations with clinical outcomes, efficiency benchmarks, and patient-centered measures.

## Introduction

Efficient inpatient transport is a critical component of hospital operations, linking clinical departments, diagnostic units, and treatment areas (1). Delays, communication gaps, and manual dispatch processes can disrupt clinical workflow, prolong waiting times, and affect overall patient experience. As hospitals grow in size and complexity, the demand for timely and coordinated transport has increased substantially, placing additional pressure on logistics teams and support departments (1). Inpatient transport is inherently complex due to the need to coordinate multiple departments, manage patients with varying acuity levels, and respond to urgent clinical needs. Limited transport staff, unpredictable demand, and reliance on manual task allocation can result in delays, miscommunication, and workflow disruptions, ultimately affecting patient safety and experience (1).

Many institutions have attempted to address these challenges by introducing digital or partially automated transport systems. However, most existing solutions rely on fragmented communication tools or manual task allocation, and few offer real-time tracking, automatic dispatching, or integration with the electronic medical record (EMR) (2). These limitations make it difficult to monitor performance, manage workload, or identify inefficiencies at scale (3). Although several hospitals have introduced digital or partially automated transport systems, many rely on fragmented communication tools or manual dispatch processes. These systems often lack real-time tracking, automated task assignment, or integration with electronic medical records, making it difficult to monitor efficiency, balance workload, and optimize resource allocation at scale (3).

With the rapid expansion of 5G networks, and artificial intelligence (AI), there is growing interest in applying intelligent logistics platforms to hospital transport management (4). Such systems have the potential to streamline coordination, improve transparency, support data-driven decision-making, and ultimately enhance the quality and safety of patient movement. Yet, despite increasing adoption, comprehensive evaluations of fully integrated intelligent transport platforms remain limited (5). Addressing these operational challenges requires a system that combines real-time coordination, automated assignment, and integration with clinical data. Intelligent transport platforms leveraging AI and 5G-enabled IoT technologies offer the potential to streamline workflows, enhance transparency, and improve both staff efficiency and patient safety, capabilities that our implemented system seeks to deliver.

In this context, we developed and implemented an intelligent inpatient transportation management system that combines EMR integration with 5G IoT positioning, automated task assignment, and a real-time monitoring dashboard. This study presents a retrospective, descriptive evaluation of the system's implementation in a large tertiary hospital, focusing on transport process characteristics, safety indicators, workforce utilization patterns, and patient and staff satisfaction.

## Materials and methods

We conducted a retrospective, descriptive review of all inpatient transportation requests at Quzhou Hospital, China, between June 2022 and September 2024. The intelligent transportation platform was launched in June 2022, and system performance data have been continuously collected since implementation.

Quzhou Hospital is a large tertiary care center with approximately 1,200 inpatient beds and over 45,000 admissions annually. The hospital serves a diverse patient population and encompasses multiple specialized clinical departments, including internal medicine, surgery, cardiology, oncology, and critical care units. Daily operations involve complex coordination of diagnostic, surgical, and therapeutic services across several buildings and floors, making timely and efficient inpatient transport a critical operational priority.

Inpatient transport at Quzhou Hospital faces multiple operational challenges. Transport tasks must be coordinated across several buildings and floors, often under high patient volume and variable urgency. Limited transport staff, peak-hour workload surges, and reliance on manual coordination prior to system implementation contributed to delays, task overlap, and occasional miscommunication. These challenges underscored the need for a more intelligent, automated platform to optimize task assignment, monitor workload, and ensure timely patient movement

The inpatient transportation system was developed using a modular design consisting of three core components: (1) a Dispatching system, used by the clinical units to submit transport requests; (2) a Smart Dispatching or “Grabbing” System which automatically assigns tasks based on predefined rules and real-time worker availability; and (3) a real-time data system which monitors transport status, workload, and performance indicators.

**Figure 1:**
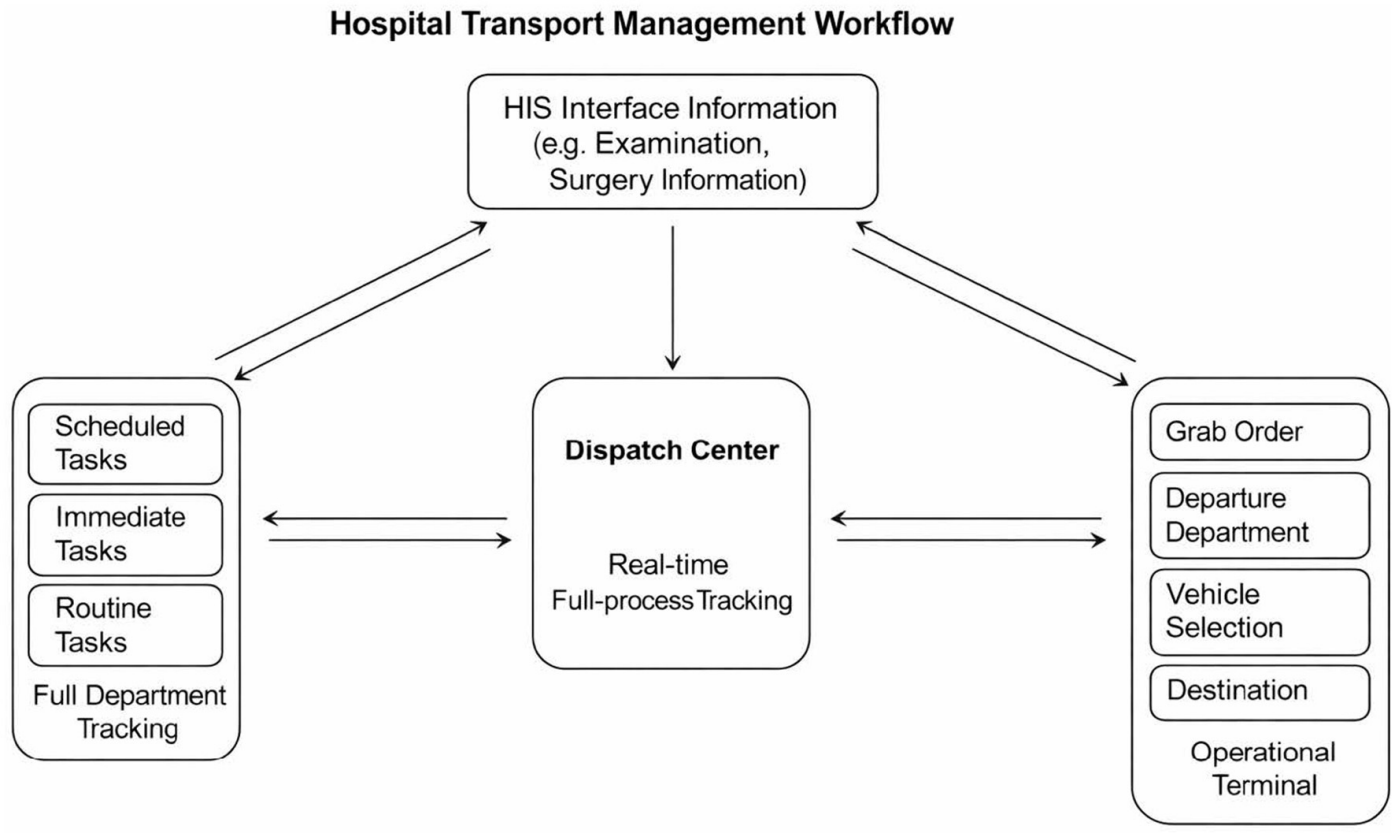
Simple working diagram of the Didi-style transportation management platform for inpatients. This diagram illustrates the step-by-step operation of the hospital transport management system. Transport requests are generated via the hospital information system (HIS) interface, including examination and surgery information. Requests are processed by the central dispatch center, which provides real-time full-process tracking. Tasks are categorized into scheduled, immediate, or routine, and can be assigned automatically or grabbed by staff through the operational terminal. The system tracks departure department, vehicle selection, and destination, enabling continuous monitoring, full-department tracking, and performance evaluation.

The platform integrates directly with the hospital's electronic medical record (EMR) system and captures transport-related information at the time an order is generated (Figure 1). Additional technical specifications and workflow details are provided in the Supplementary Appendix.

The study objectives were to describe the operational feasibility, adoption, and performance metrics of the intelligent inpatient transportation system. Formal hypothesis testing or inferential analyses were not conducted, as the retrospective, single-site design and limited pre-implementation data preclude causal inference. Given the retrospective, descriptive design and absence of a comparator group, this study was not intended to support causal inference or formal effectiveness evaluation.

### Key technological features

Transport tasks are generated automatically when the ward initiates a request for surgical or diagnostic movement. A rule engine determines task allocation based on factors such as urgency, personnel qualifications, inspection-specific requirements, current workload, and optimized transport paths. The algorithm dynamically prioritizes tasks and assigns them to available staff in real time, while also allowing worker-initiated task “grabbing” within predefined rules. Full details of the rule engine and technological architecture are included in the Supplementary Appendix.

The platform was designed to support operational efficiency, facilitate coordination of patient and equipment transport across hospital units, and provide data for performance monitoring based on workload, transport difficulty, and distance. Workforce management efficiencies and long-term cost considerations were additional implementation goals.

We extracted the following variables for all transport requests during the study period: time in minutes to hospital destinations, operating costs savings, total order volume including online and offline volumes, satisfaction rates, time differences in minutes in inspection times for different tasks, time difference in minutes between task initiation and completion, and time difference in minutes between different buildings/wards. Adverse events and patients' complaints were recorded. Adverse events were defined as any transport-related incident with potential or actual harm to patients, including falls, misidentification, or equipment-related issues. Complaints were defined as any patient or staff-reported dissatisfaction specifically related to transportation encounters. Both adverse events and complaints were captured through the hospital's routine incident reporting system, transport platform logs, and patient/staff feedback channels. Reported events were verified by departmental supervisors during routine quality assurance reviews. We note that reporting relied partly on manual entry and may underestimate the true frequency of safety events.

Satisfaction surveys were developed as part of the hospital's quality improvement program and underwent internal validation in June 2022 through pilot testing, expert review, and iterative refinement. Surveys were completed by patients and clinical staff involved in inpatient transportation encounters. Items were scored on a 0–100 scale, with higher scores indicating greater satisfaction. The initial survey included nine items; in July 2024, it was updated to include seven domains, service attitude, service quality and efficiency, accuracy and timeliness of specimen and drug delivery, appearance, labor discipline, standardized service, and overall satisfaction, while maintaining consistent administration and scoring procedures. Survey details are provided in the Supplementary Appendix.

### Statistical analysis

Continuous variables are presented as mean ± standard deviation (SD), and categorical variables as frequencies and percentages. Analyses were descriptive only, consistent with the focus on characterizing system implementation and operational performance. Missing data were reported and excluded from summary statistics. Statistical analyses were performed using standard spreadsheet and statistical software.

### Ethical considerations

No Institutional Review Board (IRB) approval was sought, as the project did not involve human subject research requiring consent. The study complied with ethical standards for quality improvement initiatives and data privacy protections.

## Results

### Satisfaction outcomes

The first complete transportation performance report was obtained in October 2022. During the early testing phase, the overall satisfaction score was 69.68%, with the lowest rating observed for timeliness of delivery (56.66%) and the highest for appearance (86.01%) (Table 1). Following system adjustments and optimization, the overall satisfaction score increased to 90% in September 2024, with particularly strong ratings in service quality and timeliness (Table 2). A detailed month-by-month breakdown of satisfaction scores from 2022 to 2024 is provided in the Supplementary Appendix. The number of transportation staff remained stable over the study period (range: 28–32; mean: 30), while the proportion of online-based workers increased from 66% in 2022 to 83% in 2024.

**Table 1:** Quzhou people's hospital transportation work satisfaction survey form in october 2022.

**Survey Items**	**Satisfaction rate (*n*= 158), mean**
Dress appropriately and wear a name tag	86.01
Timely delivery and correct preparation	56.66
Pick it up promptly after the inspection is completed	62.08
Whether you bring the correct vehicle according to the reservation requirements	70.52
Whether to check the patient's name, bed number and examination items	74.23
Send the patient to check whether the transportation is strictly in accordance with the requirements, and patiently explain the work	65.49
Don't hang out or chat in the ward	75.73
Do not push the envelope and meet temporary and patient needs.	64.21
Seat belts must be used when transporting patients in wheelchairs and flat cars to ensure patient safety.	72.17
Overall score.	69.68

**Table 2:** Quzhou People's Hospital Transportation Work Satisfaction Survey Form in September 2024.

**Survey Items**	**Satisfaction rate (*n* = 325), mean**
**Service attitude**	
Proactive and enthusiastic	2.69
Considerate service	3.56
Service with a smile	2.72
**Service quality and work efficiency**	
No errors, incorrect specimen delivery, or specimen loss	6.41
No patients fell off the bed or were lost during the examination.	6.45
Cooperate with the hospital's Didi logistics work to ensure that 90% of employees are online	5.36
**Accuracy and timeliness of specimen and drug delivery for inspection**	
Ensure timely delivery of inspections, and emergency treatment is guaranteed to take 10–15 min	4.43
Accurate and timely delivery of drugs for inspection	4.55
Accurate and timely delivery of specimens for inspection	4.59
No shirk, meeting clinical and patient needs	4.54
Ensure timely and accurate submission of patients for examination	4.47
Timely pick up after examination	8.67
Master the use of carts and wheelchairs to ensure patient safety	4.59
**Appearance**	
Dress appropriately and wear a name tag	1.98
Wear a mask or related protective measures as required	2.85
**Labor discipline**	
Abide by labor discipline and come to get off work on time	4.66
Do not do private work during working hours, do not chat on the phone, do not play and fight among employees, and do not eat snacks during working hours	4.57
**Standardized services**	
Specimen baskets are used for specimen submission and drug transportation, and special plastic bags are used for static dispensing of medicines.	1.99
Seat belts must be used when transporting patients in wheelchairs, flatbeds, and hospital beds to ensure patient safety.	2.85
**Patient and department satisfaction**	
No complaints (no points will be awarded if there are complaints)	8.99
Overall score.	90.0

### Transport volume and completeness of data

Between August 31 and September 30, 2024, a total of 15,443 online transportation requests were recorded. Of these, 503 requests lacked essential time data and were excluded, leaving 14,940 complete requests for analysis.

Across the full implementation period (January 2023–September 2024), online transport orders increased steadily, with the difference between online and offline requests widening from 6,890 in January 2023 to 13,711 by September 2024 (Supplementary Appendix).

### Department-level transport times

Mean transport times (task initiation to task completion) varied across departments (Table 3). The fastest average times were observed in ophthalmology (34.78 ± 20.70 min) and gynecology (34.94 ± 28.03 min). In contrast, emergency medicine (98.64 ± 120.37 min) and hand and foot surgery/trauma orthopedics (138.08 ± 170.06 min) had the longest mean durations.

**Table 3:** Time differences according to department categories.

**Department**	**Time difference between task initiation and completion**	**Time difference between task appointment and delivery**
	**Mean** ±**SD**	**Mean** ±**SD**
Breast and thyroid surgery, *n* = 35	49.64 ± 42	−31.02 ± 238.46
Obstetrics, *n* = 399	52.09 ± 77.48	11.47 ± 81.85
Pediatrics, *n* = 17	50.5 ± 51.61	−26.68 ± 139.71
General medicine, *n* = 85	50.39 ± 38.76	18.59 ± 46.7
Joint surgery, *n* = 545	88.59 ± 94.02	−29.69 ± 105
Endocrine and metabolic diseases, *n* = 200	48.26 ± 55.79	9.76 ± 78.52
Wound repair, *n* = 88	45.49 ± 38.47	−4.52 ± 35.71
Dentistry, *n* = 1	47.77 ± Not estimated	71.37 ± Not estimated
Respiratory and critical care medicine, *n* = 980	54.73 ± 84.88	2.65 ± 86.37
Gynecology, *n* = 19	34.94 ± 28.03	13.5 ± 38.03
Rehabilitation medicine, *n* = 323	91.44 ± 120.06	−15.18 ± 173.34
Cardiothoracic surgery, *n* = 268	46.75 ± 38.58	−1.89 ± 45.24
Cardiovascular medicine, *n* = 1,793	51.93 ± 61.18	−7.94 ± 123.08
Emergency room, *n* = 339	98.64 ± 120.37	−34.91 ± 147.07
Infectious diseases, *n* = 39	42.93 ± 46.27	−19.2 ± 243.38
Hand and foot surgery, trauma orthopedics, *n* = 1,204	138.08 ± 170.06	−44.86 ± 85.73
Urology, *n* = 219	51.97 ± 43.21	−1.83 ± 124.32
Gastroenterology, *n* = 648	50.72 ± 64.11	7.06 ± 64.23
Hernia and pediatric surgery, *n* = 92	40.79 ± 37.86	4.13 ± 50.73
Pain medicine, *n* = 34	73.25 ± 153.04	50.92 ± 176.36
ophthalmology, *n* = 14	34.78 ± 20.7	22.19 ± 56.32
Neurology, *n* = 2,521	69.62 ± 123.2	−35.32 ± 177.04
Neurosurgery, *n* = 854	94.28 ± 106.96	−25.01 ± 119.02
Colorectal surgery, *n* = 163	41.68 ± 44.51	5.54 ± 51.47
Otolaryngology, *n* = 37	57.3 ± 55.02	25.37 ± 58.15
Anal and pelvic floor surgery, *n* = 111	66.39 ± 203.49	28.16 ± 211.37
Hepatobiliary surgery, *n* = 547	51.63 ± 50.56	−6.88 ± 117.01
Nephrology, *n* = 492	53.01 ± 56.18	6.87 ± 68.95
Medical oncology, *n* = 217	50.75 ± 44.9	8.25 ± 65.4
Radiation oncology, *n* = 210	48.69 ± 46.69	17.16 ± 69.45
Gastric surgery, *n* = 286	56.78 ± 74.48	5.47 ± 52.09
Pancreatic diseases, *n* = 200	46.46 ± 37.44	0.65 ± 87.25
Spine surgery, *n* = 884	106.74 ± 113.19	−26.51 ± 67.07
Hematology, *n* = 370	48.98 ± 52.28	11.37 ± 66.88
Vascular surgery, *n* = 431	53.36 ± 54.87	−5.62 ± 67.96
Rheumatology, *n* = 2 75	42.78 ± 36.17	4.61 ± 84.06
Grand total, *n* = 14,940	**70.34** **±101.56**	–**13.73** **±118.06**

Bold values indicate the maximum and minimum mean values in each category.

Differences between scheduled and actual delivery times, reflecting earlier-than-scheduled arrivals, were most pronounced in hand and foot surgery/trauma orthopedics (−44.86 ± 85.73 min) and the emergency department (−34.91 ± 147.07 min). These observations are presented as descriptive metrics reflecting operational performance rather than evaluative comparisons.

### Ward-level transport times

Transport times also varied considerably by ward (Table 4). Wards 5–8–1A and 6–6–2 had the longest mean times between task initiation and completion (134.33 ± 93.67 min and 130.50 ± 164.98 min, respectively). Conversely, the shortest average times were recorded in wards 5–5–2 (38.11 ± 29.34 min) and 5–10–1 (43.45 ± 35.06 min).

**Table 4:** Time differences according to ward categories.

**Ward**	**Time difference between task initiation and completion**	**Time difference between task appointment and delivery**
	**Mean** ±**SD**	**Mean** ±**SD**
**1−2−1**, ***n*** **=** **339**	98.64 ± 120.37	−34.91 ± 147.07
3−10−1, *n* = 119	50 ± 36.56	11.5 ± 49.38
3−3−1, *n* = 103	46.27 ± 41.86	10.58 ± 77.2
3−4−1, *n* = 337	47.14 ± 39	−4.16 ± 103.53
3−5−1, *n* = 54	45.71 ± 46.36	24.89 ± 59.81
3−6−1, *n* = 115	52.52 ± 51.62	10.24 ± 70.1
3−8−1, *n* = 324	91.18 ± 119.97	−15.13 ± 173.07
5−10−1, *n* = 215	43.45 ± 35.06	−0.57 ± 51.25
5−10−2, *n* = 94	62.36 ± 51.51	−11.3 ± 154.07
5−11−1, *n* = 214	53.69 ± 51.14	4.61 ± 49.21
5−11−2, *n* = 214	49.69 ± 78.67	6.49 ± 77.88
5−12−2, *n* = 454	53.26 ± 54.19	−3.82 ± 65.35
5−13−2, *n* = 41	49.25 ± 46.89	−40.8 ± 223.86
5−3−1, *n* = 232	44.98 ± 44.13	17.67 ± 39.88
5−5−1, *n* = 169	61.63 ± 106.71	3.14 ± 116.46
5−5−2, *n* = 15	38.11 ± 29.34	16.71 ± 42.06
5−6−1, *n* = 905	105.86 ± 105.12	−27.79 ± 69.41
5−6−2, *n* = 349	53.37 ± 88.57	−2.9 ± 50.98
5−7−1, *n* = 662	131 ± 157.91	−43.37 ± 99.19
5−7−2, *n* = 645	89.59 ± 93.98	−25.84 ± 96.4
5−8−1A, *n* = 108	134.33 ± 93.67	−50.07 ± 77.73
5−8−1, *n* = 145	108.72 ± 58.33	−43.34 ± 62.02
5−8−2, *n* = 610	87.94 ± 144.08	−14.18 ± 161.91
5−9−1, *n* = 187	45.52 ± 40.2	−1.51 ± 72.1
5−9−2, *n* = 249	54.35 ± 54.6	3.67 ± 90.77
6−10−1, *n* = 315	56.17 ± 76.79	−0.09 ± 73.5
6−10−2, *n* = 312	44.72 ± 43.6	14.58 ± 57.7
6−11−1, *n* = 137	52.09 ± 64.76	12.34 ± 85.52
6−11−2, *n* = 427	44.2 ± 39.23	6.7 ± 73.81
6−12−2, *n* = 148	51.52 ± 65.99	2.31 ± 128.94
6−13−2, *n* = 504	52.78 ± 51.95	6.61 ± 68.03
6−3−1, *n* = 17	50.5 ± 51.61	−26.68 ± 139.71
6−3−2A, *n* = 10	55.3 ± 50.73	16.92 ± 48.35
6−4−1, *n* = 494	52.42 ± 57.02	−6.85 ± 88.97
6−4−2, *n* = 1,210	49.9 ± 56.49	−5.74 ± 128.9
6−5−1, *n* = 151	115.2 ± 216.14	16.03 ± 104.18
6−5−2, *n* = 223	57.16 ± 55.42	−4.43 ± 136.4
6−6−1, *n* = 150	45.87 ± 61.99	9.16 ± 83.31
6−6−2, *n* = 468	130.5 ± 164.98	−44.22 ± 68.15
6−7−1, *n* = 1,247	69.59 ± 133.84	−62.6 ± 220.58
6−7−2, *n* = 1,218	69.21 ± 112.9	−9.64 ± 113.64
6−8−1, *n* = 201	46.39 ± 65.04	2.8 ± 91.2
6−8−2, *n* = 184	67.1 ± 115.26	−3.45 ± 81.75
6−9−1, *n* = 331	56.29 ± 68.09	5.74 ± 75.28
6−9−2, *n* = 280	50.34 ± 88.95	2.31 ± 96.98
7−2−1, *n* = 38	43.68 ± 46.66	−18.9 ± 246.64
7−3−1, *n* = 1	14.68 ± Not estimated	−30.37 ± Not estimated
7−4−1, *n* = 1	70.87 ± Not estimated	0.48 ± Not estimated
Grand total, *n* = 14,940	**70.34** **±101.56**	−13.73 ± 118.06

Bold values indicate the maximum and minimum mean values in each category.

Differences between scheduled and actual delivery times followed a similar pattern, with wards 6–7–1 (−62.60 ± 220.58 min) and 5–8–1A (−50.07 ± 77.73 min) showing the largest negative time differences.

### Safety events and complaints

No incidents of incorrect patient identification occurred during the study period. One fall-from-bed event was reported in 2023. System-related delays accounted for 6.7% of all recorded complaints, most involving transports to or from the emergency department.

## Discussion

Patient transportation inside the hospital is an essential part of day-to-day clinical operations. Whether the request is routine, urgent, or truly emergent, the process needs to be efficient, coordinated, and safe (6). Although intrahospital transport can be divided into the pre-transport, active transport, and post-transport phases, there are still no widely accepted standards or guidelines for how these steps should be organized within the hospital. In many institutions, variability in infrastructure, staffing, and workflow contributes to inconsistent transport times and occasional safety concerns (7). These challenges were part of the motivation to develop an integrated, technology-based platform that could bring more structure and accountability to the process (7).

In this study, we evaluated the performance of a fully digital inpatient transport system that combines information technology, 5G IoT positioning, automatic dispatching, and real-time monitoring. The system was applied across all hospital departments, and the data over time give a broad picture of how transportation performance changed after implementation. As expected, transport times (from task initiation to task completion) differed considerably between specialties. Departments such as ophthalmology and gynecology had much shorter average transport times, which may reflect the relative simplicity of their transport needs and the absence of extensive pre-procedure checklists. In contrast, services involving surgical patients, such as trauma, hand and foot surgery, neurosurgery, and other complex surgical units, showed longer transport times. These cases typically require additional verification steps, preparation, and coordination among staff, all of which add time to the process. Prior work has shown that surgical checklists and pre-procedure requirements can prolong preparation time if teams are not fully aligned or trained (8, 9).

Transport to the emergency department showed an interesting pattern. The overall transport time was longer, which is not surprising given the unpredictability and acuity of emergency cases. Staff often need to remove or replace personal protective equipment or gather additional support personnel before moving certain patients. Despite this longer preparation time, the difference between scheduled and actual delivery times was shorter, suggesting that once the request is fully processed, transport to the emergency department tends to happen quickly. This is consistent with the priority usually given to emergency cases and with the broader issue of emergency department crowding, which can affect throughput and increase pressure to move patients rapidly (10, 11).

Differences between scheduled and actual delivery times, including negative values indicating earlier-than-scheduled arrivals, were most prominent in hand and foot surgery/trauma orthopedics (−44.86 ± 85.73 min) and the emergency department (−34.91 ± 147.07 min). These metrics reflect operational performance rather than clinical outcomes: negative values may indicate proactive scheduling, prioritization of high-acuity cases, or variability in patient readiness. Overall, the combination of initiation-to-completion time and appointment-to-delivery difference provides insight into workflow efficiency, responsiveness, and department-level coordination within the hospital transport system (11).

Patient safety remains the most important aspect of any transport system, and several findings in our study are reassuring. Satisfaction scores improved steadily over time, from 69.7% early in the testing phase to about 90% in late 2024. The highest satisfaction scores were observed in service quality, work efficiency, and timeliness of delivery, which coincided with system automation and coordination between departments. During the early implementation phase, lower scores for timely delivery prompted targeted adjustments, including realignment of transporter shifts to match peak-hour demand, optimization of automated task allocation rules, and refinement of staff notification alerts. These changes coincided with subsequent improvements in timeliness and overall satisfaction

Importantly, we observed no adverse events caused by wrong patient identification. Considering that more than 460,000 transport requests have been handled since the system went live, the absence of identification errors suggests that the integration with the International Patient Safety Goals was effective. Other safety-related events were rare; only one fall from bed occurred in 2023, and most complaints (6.7%) involved delays during peak hours, mainly related to emergency transport. These issues may be reduced through closer monitoring of peak-hour demand and dynamic adjustment of staffing.

The platform also offered several operational advantages. It automated most aspects of the process, tracked transporters in real time using GPS, and provided a dashboard that allowed supervisors to identify delays, reassign staff, and monitor usage patterns. The system works in a way that is conceptually similar to common ride-share applications, assigning transporters based on availability, proximity, and priority. These features make the workflow more transparent and easier to manage. The increasing shift from offline to online task handling, from 66 to 83% over two years, also suggests growing adoption and trust in the system among staff.

Nevertheless, there are limitations worth noting. Pre-implementation data were incomplete; although about 5,000 transports were identified from before the system went live, only 76 had fully usable data. As a result, the study does not provide formal before-and-after comparisons, and any apparent differences over time should not be interpreted as performance gains; findings are presented descriptively to reflect operational performance. This limitation of data is consistent with other intra-hospital transport system utilized by Hains et al. (12) which reported similar but bit different results compared to our results. Additionally, compared to manual or semi-digital transport systems reported in the literature, our descriptive metrics suggest generally shorter transport times and improved workflow coordination, while recognizing the limitations of indirect comparison (12). We also did not measure actual transport distances, which would have helped interpret department- and ward-level differences more precisely. Reporting of adverse events depended partly on manual input by transport staff, which may underestimate certain categories of incidents, such as minor delays or near-miss events. Therefore, while serious safety breaches were not observed, these results should be interpreted as descriptive indicators of operational safety rather than definitive measures of risk reduction. Finally, time differences might be further improved if patients are fully prepared when transporters arrive, which is an area that may require additional coordination and patient-family education. Accordingly, findings should be interpreted as descriptive indicators of operational feasibility, system adoption, and process performance rather than evidence of causal effectiveness.

## Conclusion

We present a full descriptive review of our intelligent inpatient transportation system and its performance across a large tertiary hospital. The platform proved to be efficient, safe, and well-accepted by both patients and staff, and it was able to handle a high volume of transport requests without compromising service quality. While the study does not include formal comparative or inferential analyses, the findings provide insights into system functionality, workflow integration, and user satisfaction. Future work should focus on collecting more robust clinical outcomes, including detailed tracking of adverse events, and on conducting direct comparisons between pre-implementation and post-implementation to better characterize the system's performance and role within hospital transport workflows.

## Data Availability

The raw data supporting the conclusions of this article will be made available by the authors, without undue reservation.
