# Supplementary material for: Evaluation of an intelligent inpatient transportation system in a large tertiary hospital: a full implementation case review
Source: Front Public Health. 2026 Mar 18;14:1758228. doi: 10.3389/fpubh.2026.1758228 (PMC13039065; doi:10.3389/fpubh.2026.1758228)
Supplement: Supplementary file 1 [file Data_Sheet_1.docx]

**Supplementay Appendix**

|  | **Page** |
| --- | --- |
| **Context P1:** The workflow and design of the system platform | 2 |
| **Context P2:** The key technologies of the system platform. | 5 |
| **Supplementary Table 1:** Detailed Quzhou People’s Hospital Transportation Work Satisfaction Survey Form in October 2022. | 6 |
| **Supplementary Table 2:** Detailed Quzhou People’s Hospital Transportation Work Satisfaction Survey Form in September 2022. | 7 |
| **Supplementary Table 3:** Overall Satisfaction stratified by month. | 9 |
| **Supplementary Table 4:** Online and offline order volume and personal costs. | 10 |
| **Supplementary Table 5:** Detailed Shipment volumes. | 11 |

**The workflow and design of the system platform.**

**Design:** The system platform connects to the hospital HIS system, opens up the doctor's order system, appointment system, and queuing system, integrates examination departments, operating rooms, inpatient wards, patients, delivery workers, and other medical links to generate task orders, and through the establishment of Didi's order-grabbing and intelligent order-dispatching models, workers can grab orders, and the system automatically and intelligently dispatches orders for tasks that no one can grab; the Internet of Things technology is used to achieve real-time positioning of all delivery personnel. In the process of grabbing and dispatching orders, the system combines the location of the delivery personnel, current work status, current delivery point, expected route, expected delivery time, etc., and can realize AI intelligent algorithms such as nearby order dispatching, smooth order dispatching, group order dispatching, and connection order dispatching to improve transportation scheduling efficiency; a service evaluation system is established. After the transportation service is completed, the patient or nurse will evaluate the service to achieve performance appraisal based on workload and service quality.

**Workflow:** The system platform for inpatients consists of three parts: the dispatching system, the smart dispatching system/order grabbing system, and the real-time data system; the hardware involves: the dispatching center PC, the task sending computer used by the nurse station, the order receiving/order grabbing mobile phone used by the workers, the 5G signal card, the exclusive cloud server, etc. The platform can be seamlessly connected with the hospital's His system, and can accurately collect data related to inspection and surgical tasks in the first place.

- Dispatching system: According to the requirements of different tasks, a rule engine is set up for rule configuration, such as the rules for different transport personnel to perform tasks; the rules for task allocation and the real-time status of transport workers, etc. The combination of rules and algorithms ultimately realizes the supervision of task execution.
- Smart dispatching system/Didi grabbing system: Task execution monitoring is performed through the self-developed App on smartphones. When workers automatically grab orders online, no one grabs orders or other specific situations occur, the dispatching center will dispatch tasks to a certain worker according to the dispatching algorithm. The worker follows the process of scanning the code to pick up people, transport, and deliver to the destination, and then scans the code to complete the task.
- Real-time data system: The IoT data exchange and sharing platform is used to collect in-hospital business data, smart building system data, map data, positioning data, IoT sensor data, etc., including a unified map system, positioning system, process management system, unified alarm system, etc., to share data and provide standardized services. For example, the order-taking system feeds back its own location information and task progress to the background in real time, realizes the full process tracking of the service, and provides a basis for later data analysis (**Supplement Figure 1**).


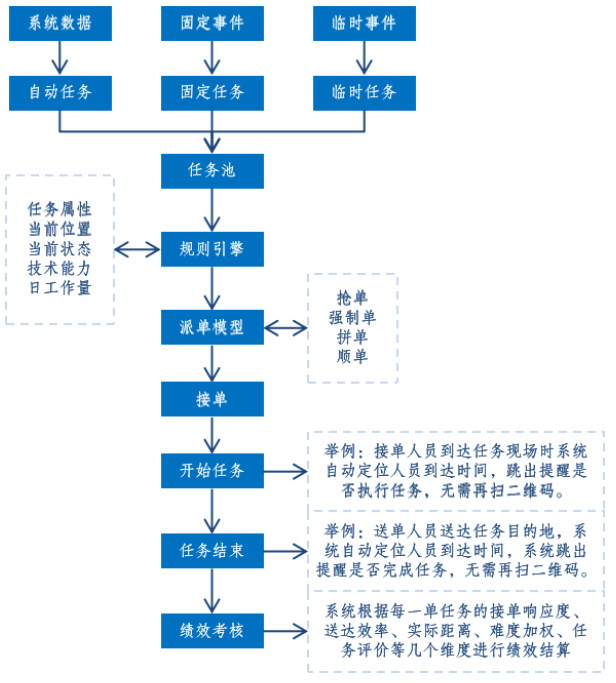


**Supplement Figure 1:** Workflow of drip-type transportation of inpatients

**Key technologies.**

##### Task generator: Transport tasks generally refer to the work initiated by the ward according to medical requirements, including surgical transport, examination transport, etc. According to the doctor's order, combined with the time requirements of the appointment system, and the real-time information of the queuing system, the system automatically generates a task list. The hospital uses secure internal and external network interface technology to synchronize the appointment examination, immediate examination and surgery HIS information related to the transport service directly to the transport system and enter the task pool. At the same time, read the nature of the transport service, such as appointment task, immediate task or regular task.

##### Rule engine design: According to the requirements of different tasks, the rule engine is used to configure rules, such as the inspection path requirements of a security guard; the rules for different personnel to perform tasks; the rules for task allocation, etc. The combination of rules and algorithms ultimately achieves the controllability of task execution. Rules are configured for each type of transportation task, including personnel type, skill level, priority, distance priority, timeliness, fixed, temporary, automatic, group order, order order, grab order, etc.

##### Order dispatch model: calculation rules for dispatching, grabbing, receiving, delivering, combining, and sorting orders.

##### IoT base station: unified construction of hospital IoT network, eliminating the phenomenon of "one application, one network", must be able to fully support all IoT applications in hospitals in the future. Support data collection of various IoT sensors in the medical IoT, such as data collection of wearable vital signs equipment, working condition data collection of medical equipment, temperature and humidity data collection of medical environments, positioning data collection of various IoT terminals, etc. Build a unified IoT data exchange and sharing platform, including visual management of IoT network equipment, unified management of IoT terminals, unified electronic map engine, unified positioning engine, unified message engine, unified IoT data warehouse, unified IoT data interface standard, etc.

| Survey Items | Max Score |
| --- | --- |
| Dress appropriately and wear a name tag | 100 |
| Timely delivery and correct preparation | 100 |
| Pick it up promptly after the inspection is completed | 100 |
| Whether you bring the correct vehicle according to the reservation requirements | 100 |
| Whether to check the patient’s name, bed number and examination items | 100 |
| Send the patient to check whether the transportation is strictly in accordance with the requirements, and patiently explain the work | 100 |
| Don’t hang out or chat in the ward | 100 |
| Do not push the envelope and meet temporary and patient needs. | 100 |
| Seat belts must be used when transporting patients in wheelchairs and flat cars to ensure patient safety. | 100 |

**Supplementary Table 1:** Detailed Quzhou People’s Hospital Transportation Work Satisfaction Survey Form in October 2022.

Each item is scored from zero to 100, where zero indicates no satisfaction at all, and 100 indicates highest satisfaction rate.

| Survey Items | Max Score |
| --- | --- |
| Service attitude | |
| Proactive and enthusiastic | 3 |
| Considerate service | 4 |
| Service with a smile | 3 |
| Service quality and work efficiency | |
| No errors, incorrect specimen delivery, or specimen loss | 7 |
| No patients fell off the bed or were lost during the examination. | 7 |
| Cooperate with the hospital’s Didi logistics work to ensure that 90% of employees are online | 6 |
| Accuracy and timeliness of specimen and drug delivery for inspection | |
| Ensure timely delivery of inspections, and emergency treatment is guaranteed to take 10-15 minutes | 5 |
| Accurate and timely delivery of drugs for inspection | 5 |
| Accurate and timely delivery of specimens for inspection | 5 |
| No shirk, meeting clinical and patient needs | 5 |
| Ensure timely and accurate submission of patients for examination | 5 |
| Timely pick up after examination | 10 |
| Master the use of carts and wheelchairs to ensure patient safety | 5 |
| Appearance | |
| Dress appropriately and wear a name tag | 2 |
| Wear a mask or related protective measures as required | 3 |
| Labor discipline | |
| Abide by labor discipline and come to get off work on time | 5 |
| Do not do private work during working hours, do not chat on the phone, do not play and fight among employees, and do not eat snacks during working hours | 5 |
| Standardized services | |
| Specimen baskets are used for specimen submission and drug transportation, and special plastic bags are used for static dispensing of medicines. | 2 |
| Seat belts must be used when transporting patients in wheelchairs, flatbeds, and hospital beds to ensure patient safety. | 3 |
| Patient and department satisfaction | |
| No complaints (no points will be awarded if there are complaints) | 10 |

**Supplementary Table 2:** Detailed Quzhou People’s Hospital Transportation Work Satisfaction Survey Form in September 2024.

| Month-Year | | | | | | | | | | | | | |
| --- | --- | --- | --- | --- | --- | --- | --- | --- | --- | --- | --- | --- | --- |
| Year | Month | January | Feb | March | April | May | June | July | August | Sept | October | November | December |
|  | 2022 | -- | -- | -- | -- | -- | -- | -- | -- | -- | 69.67 | 89 | 71 |
|  | 2023 | 67 | 62 | 65 | 64.3 | 68 | 79.43 | 79.45 | 83.99 | 73.4 | 73.94 | 82.76 | 75.47 |
|  | 2024 | 80.09 | 79.94 | 76.57 | 77.09 | 80.52 | 84.52 | 90.31 | 90.17 | 90.9 |  |  |  |

**Supplementary Table 3:** Detailed Satisfaction rates from 2022 to 2024.

| Year-Months | | January | Feb | March | April | May | June | July | August | Sept | Octo | Novem | December |
| --- | --- | --- | --- | --- | --- | --- | --- | --- | --- | --- | --- | --- | --- |
| 2022 | **people online** | -- | -- | -- | -- | -- | 20 | 20 | 20 | 20 | 20 | 20 | 20 |
|  | **people offline** | -- | -- | -- | -- | -- | 10 | 10 | 10 | 10 | 10 | 10 | 10 |
|  | **Total** | -- | -- | -- | -- | -- | 30 | 30 | 30 | 30 | 30 | 30 | 30 |
|  | **Service fee payment** | -- | -- | -- | -- | -- | 122850 | 122850 | 122850 | 122850 | 122850 | 122850 | 122850 |
| 2023 | **people online** | 19 | 20 | 16 | 16 | 20 | 20 | 25 | 22 | 20 | 22 | 22 | 25 |
|  | **people offline** | 11 | 15 | 9 | 9 | 15 | 15 | 8 | 8 | 8 | 7 | 5 | 6 |
|  | **Total** | 30 | 35 | 25 | 25 | 35 | 35 | 33 | 30 | 28 | 29 | 27 | 31 |
|  | **Service fee payment** | 125421 | 146324.5 | 104517.5 | 104517.5 | 146324.5 | 146324.5 | 137963.1 | 125421 | 117059.6 | 121240.3 | 112878.9 | 129601.7 |
| 2024 | **people online** | 27 | 27 | 27 | 27 | 27 | 25 | 25 | 25 | 25 | -- | -- | -- |
|  | **people offline** | 6 | 6 | 4 | 4 | 4 | 5 | 5 | 5 | 5 | -- | -- | -- |
|  | **Total** | 33 | 33 | 31 | 31 | 31 | 30 | 30 | 30 | 30 | -- | -- | -- |
|  | **Service fee payment** | 137963.1 | 137963.1 | 129601.7 | 129601.7 | 129601.7 | 125421 | 125421 | 125421 | 125421 | -- | -- | -- |

**Supplementary Table 4:** Online and offline order volume, number of employees and personal costs.
***Note:*** The total number of transport personnel before October 2023 will be responsible for the examination and transportation of patients in the entire hospital, and the number of contracted positions is 35; starting from October 2023, the total number of transport personnel will be responsible for the examination and transportation of inpatients and the transportation of surgical patients, and the number of contracted positions will be 41. Moreover, the service charge was 4095$ until January 2023, after which it became 4180.7$.

| Shipment volume in 2023 | | | | | | | | | | | | |
| --- | --- | --- | --- | --- | --- | --- | --- | --- | --- | --- | --- | --- |
| Item-Month | January | Feb | March | April | May | June | July | August | Sept | October | November | December |
| Online order quantity | 9476 | 11156 | 12133 | 9812 | 9761 | 8756 | 11747 | 11916 | 11417 | 14675 | 15076 | 11522 |
| Offline order quantity | 2586 | 2243 | 2405 | 3212 | 3985 | 4523 | 3102 | 2919 | 2876 |  |  | 2017 |
| Difference | 6890 | 8913 | 9728 | 6600 | 5776 | 4233 | 8645 | 8997 | 8541 | -- | -- | 9505 |
| operating room unit | 1107 | 2780 | 3009 | 2646 | 3053 | 2716 | 3464 | 3488 | 2659 | 3151 | 2980 | 2112 |
| Total order quantity | 13169 | 16179 | 17547 | 15670 | 16799 | 15995 | 18313 | 18323 | 16952 | 17826 | 18056 | 15651 |
| Shipment volume in 2024 | | | | | | | | | | | | |
| Item-Month | January | Feb | March | April | May | June | July | August | Sept |  |  |  |
| Online order quantity | 14711 | 12253 | 16914 | 15749 | 15993 | 15531 | 15691 | 15871 | 15443 |  |  |  |
| Offline order quantity | 2113 | 2047 | 2270 | 1960 | 2153 | 1844 | 1810 | 1730 | 1732 |  |  |  |
| Difference | 12598 | 10206 | 14644 | 13789 | 13840 | 13687 | 13881 | 14141 | 13711 |  |  |  |
| operating room unit | 2854 | 2449 | 3155 | 3125 | 3193 | 2885 | 3742 | 3518 | 3109 |  |  |  |
| Total order quantity | 19678 | 16749 | 22339 | 20834 | 21339 | 20260 | 21243 | 21119 | 20284 |  |  |  |

**Supplementary Table 5:** Detailed Shipment volumes.
